# Surgical treatment of 125 cases of congenital diaphragmatic eventration in a single institution

**DOI:** 10.1186/s12893-020-00928-z

**Published:** 2020-11-04

**Authors:** Shengliang Zhao, Zhengxia Pan, Yonggang Li, Yong An, Lu Zhao, Xin Jin, Jian Fu, Chun Wu

**Affiliations:** 1 Department of Cardiothoracic Surgery, Children’s Hospital of Chongqing Medical University, Chongqing, 400014 People’s Republic of China; 2 Ministry of Education Key Laboratory of Child Development and Disorders, Chongqing, 400014 People’s Republic of China; 3 National Clinical Research Center for Child Health and Disorders (Chongqing), Chongqing, 400014 People’s Republic of China; 4 China International Science and Technology Cooperation Base of Child Development and Critical Disorders, Chongqing, 400014 People’s Republic of China; 5 Chongqing Key Laboratory of Pediatrics, Chongqing, 400014 People’s Republic of China; 6 Room 806, Kejiao Building (NO. 6 Building), No. 136, 2nd Zhongshan Road, Yuzhong District, Chongqing, China

**Keywords:** Congenital diaphragm eventration, Diaphragm plication, Thoracoscopic, Surgery

## Abstract

**Background:**

This study sought to investigate the clinical characteristics of congenital diaphragmatic eventration (CDE) and to compare the efficacies of thoracoscopy and traditional open surgery in infants with CDE.

**Methods:**

We retrospectively analyzed the clinical data of 125 children with CDE (90 boys, 35 girls; median age: 12.2 months, range: 1 h-7 years; body weight: 1.99–28.5 kg, median body weight: 7.87 ± 4.40 kg) admitted to our hospital in the previous 10 years, and we statistically analyzed their clinical manifestations and surgical methods.

**Results:**

A total of 108 children in this group underwent surgery, of whom 67 underwent open surgery and 41 underwent thoracoscopic diaphragmatic plication. A total of 107 patients recovered well postoperatively, except for 1 patient who died due to respiratory distress after surgery. After 1–9.5 years of follow-up, 107 patients had significantly improved preoperative symptoms. During follow-up, the location of the diaphragm was normal, and no paradoxical movement was observed. Eleven of the 17 children who did not undergo surgical treatment did not have a decrease in diaphragm position after 1–6 years of follow-up. The index data on the operation time, intraoperative blood loss, chest drainage time, postoperative mechanical ventilation time, postoperative hospital stay and postoperative CCU admission time were better in the thoracoscopy group than in the open group. The difference between the two groups was statistically significant (P < 0.05).

**Conclusions:**

The clinical symptoms of congenital diaphragmatic eventration vary in severity. Patients with severe symptoms should undergo surgery. Both thoracoscopic diaphragmatic plication and traditional open surgery can effectively treat congenital diaphragmatic eventration, but compared with open surgery, thoracoscopic diaphragmatic plication has the advantages of a short operation time, less trauma, and a rapid recovery. Thus, thoracoscopic diaphragmatic plication should be the first choice for children with congenital diaphragmatic eventration.

## Background

CDE is considered to result from a congenital anomaly during the formation of the pleuroperitoneal membrane, as in Bochdalek diaphragmatic hernia, but that occurs in a later stage during embryonal growth [1]. CDE is a rare pathology that occurs in 0.02 to 0.07/1000 births, affecting mostly males in 60 to 80% of cases. It accounts for 5–7% of all diaphragm diseases [2]. Because the infant ribs are horizontal and the intercostal muscles are weak, breathing movement mainly depends on the abdominal breathing of the diaphragm muscles moving up and down. Infants and children with CDE have abnormally elevated diaphragm muscles, which often leads to collapse of the affected alveoli or atelectasis, thus affecting lung ventilation and lung development. Therefore, infants and children with CDE often have symptoms such as dyspnea, repeated respiratory infections, low weight, and stunting. Severe cases may manifest as respiratory distress syndrome, seriously affecting the quality of life of children. Traditionally, diaphragmatic plication has been performed by thoracotomy or laparotomy, particularly in symptomatic, smaller children [3]. However, advancements in endoscopic surgery have allowed diaphragmatic eventration to be treated quickly and safely. Here, we present our experience with different surgical procedures to treat 125 patients with CDE.

## Methods

We retrospectively analyzed the clinical data of 125 children with congenital diaphragmatic eventration admitted to the Department of Cardiothoracic Surgery, Children's Hospital of Chongqing Medical University from January 2010 to January 2020. The Medical Research Ethics Committee of Children's Hospital Affiliated to Chongqing Medical University approved the study, and this study obtained written informed consent from the families of all children. Inclusion criteria: children with CDE and dyspnea, repeated respiratory tract infections and other symptoms. Chest X-ray, CT or gastrointestinal radiography was used to clearly diagnose diaphragmatic eventration. Exclusion criteria: children with acquired diaphragmatic eventration associated with surgery were excluded.

**Fig. 1:**
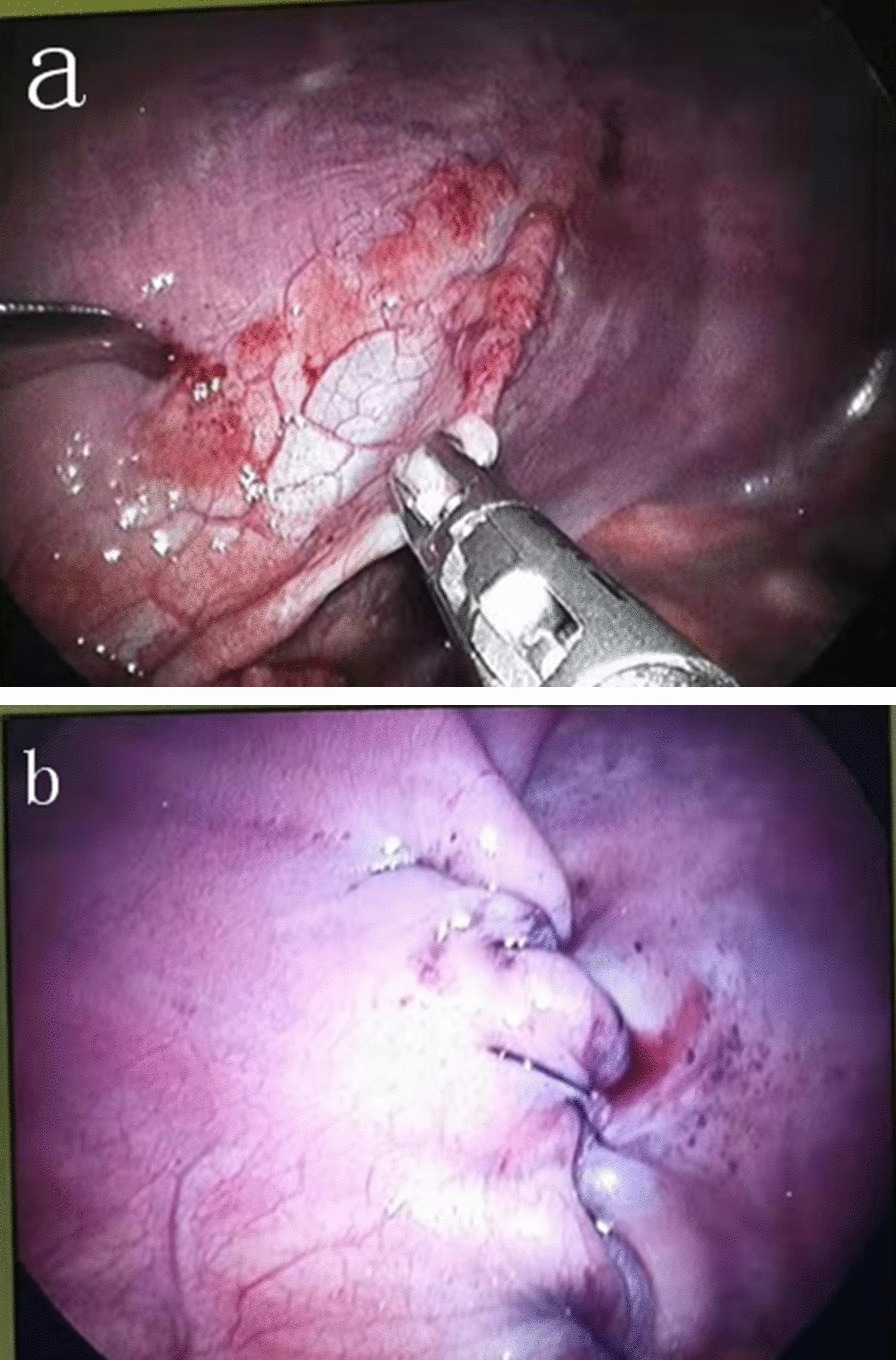
Comparison of the diaphragm before and after thoracoscopic diaphragmatic plication (n = 41). Diaphragm muscle weakness was observed before diaphragmatic plication (**a**). After diaphragmatic plication, the diaphragm was reinforced with continuous barbed wire sutures (**b**)

Open surgery group: thoracotomy was performed for those with right diaphragm eventration and laparotomy for those with left diaphragm eventration. Through the thoracoabdominal approach, we removed the weak diaphragm and used intermittent nonabsorbable sutures to ensure that the cut diaphragm had a shingled shape to strengthen the weak area of the diaphragm. Thoracoscopic group: using the three-hole method, a 5 mm trocar was placed on the lower edge of the scapula tip, and two operation holes were made in the fourth intercostal space on both sides of the trocar. Continuous barbed sutures from the outside to the inside were utilized to give the diaphragm a shingled shape in order to strengthen the diaphragm. See Fig. 1 for details.

### Statistical analyses

All the collected data were statistically analyzed using SPSS 22.0 software. The continuous variables were expressed as the mean ± standard deviation, and the classification variables were expressed as ratio columns. The comparison between the two groups was expressed by independent sample t-tests, and the count data were expressed by Fisher's precision test. The difference was statistically significant with a P value of < 0.05.

## Results

The study included 125 children diagnosed with CDE. There were 90 males (72%) and 35 females (28%), aged 1 h-7 years, with a median age of 12.2 months and a body weight of 1.99–28.5 kg (7.87 ± 4.40 kg). A total of 78 children (62.4%) had right CDE, 47 children (37.6%) had left CDE, and no children had bilateral CDE. There were 79 children with malformations in this group, mainly including 19 with congenital heart disease, 16 with congenital pulmonary dysplasia, 8 with pectus excavatum, 4 with hiatal hernia, and 3 with pectoral malformations.

The clinical symptoms of CDE were reported for 108 of 125 patients. The main symptoms of CDE in infants included cough and asthma, dyspnea, recurrent respiratory tract infections, milk refusal, vomiting, and arrhythmia. Approximately 17 patients were asymptomatic, or symptoms were accidentally discovered on routine physical examination. The clinical symptoms of CDE are shown in Table 1. All 125 cases had positive manifestations on chest X-ray, among which 39 cases (31.2%) were diagnosed by combined chest CT, 32 cases (25.6%) were diagnosed by combined chest X-ray and digestive tract radiography, and 99 cases were found to have eventration of diaphragmatic shadows. All cases were confirmed as CDE after surgery, and the position of CDE is presented in Table 2.

**Table 1:** Symptoms of congenital diaphragmatic eventration in children (n = 125)

Clinical manifestations	Open group	Thoracoscopy group	P value
Cough and asthma, difficulty breathing	37	24	0.038
Recurrent respiratory tract infection	9	6	0.298
Shortness of breath, cyanosis	10	7	0.374
Refuse milk and vomit	5	4	0.500
Arrhythmology	2	4	0.342
Asymptomatic chest X-ray findings	10	7	0.374

Rate means the probability of symptom appearance in 125 patients

**Table 2:** The position of congenital diaphragmatic eventration found during the operation (n = 108)

Diaphragmatic position	Open group	Thoracoscopy group	P value
Second front rib	4	2	0.342
Third front rib	9	6	0.297
Fourth front rib	23	41	0.005
Fifth front rib	8	3	0.107
Sixth front rib	3	1	0.311
Unclear during surgery	5	3	0.249

Rate means the probability of the position of congenital diaphragmatic eventration appearing in 125 patients

Forty-one patients underwent transthoracic diaphragm plication, and 26 patients underwent transabdominal diaphragm plication. Among them, 9 patients were diagnosed with CDE before surgery. The stomach, duodenum, spleen and part of the liver herniated into the thoracic cavity during the operation. Diaphragmatic hernia was diagnosed after the operation.

We analyzed the data of the relevant surgical indicators in the two groups. The index data on the operation time, intraoperative blood loss, chest drainage time, postoperative mechanical ventilation time, postoperative hospital stay and postoperative CCU admission time were better in the thoracoscopy group than in the open group. The difference between the two groups was statistically significant (P < 0.05). There was no statistically significant difference between the two groups in the descending distance of the diaphragm (P > 0.05). See Table 3 for details.

**Table 3:** Comparative analysis of operative-related indexes between the open group and thoracoscopy group (n = 108)

Operative related indexes	Open group (n = 67)	Thoracoscopy group (n = 41)	P value
Operation time (min)	91.17 ± 28.14	66.13 ± 18.35	0.031
Intraoperative blood loss (ml)	6.35 ± 3.41	3.46 ± 2.48	0.015
Postoperative mechanical ventilation time (day)	1.94 ± 2.13	0.87 ± 1.31	0.007
Postoperative hospital stay (day)	18.31 ± 3.07	11.23 ± 3.31	0.000
Postoperative CCU admission time (day)	4.32 ± 3.86	3.82 ± 2.38	0.000
Descending distance of diaphragm (intercostal)	3.72 ± 1.26	3.31 ± 1.33	0.534
Chest drainage time (day)	7.38 ± 3.43	4.31 ± 2.72	0.019

**Fig. 2:**
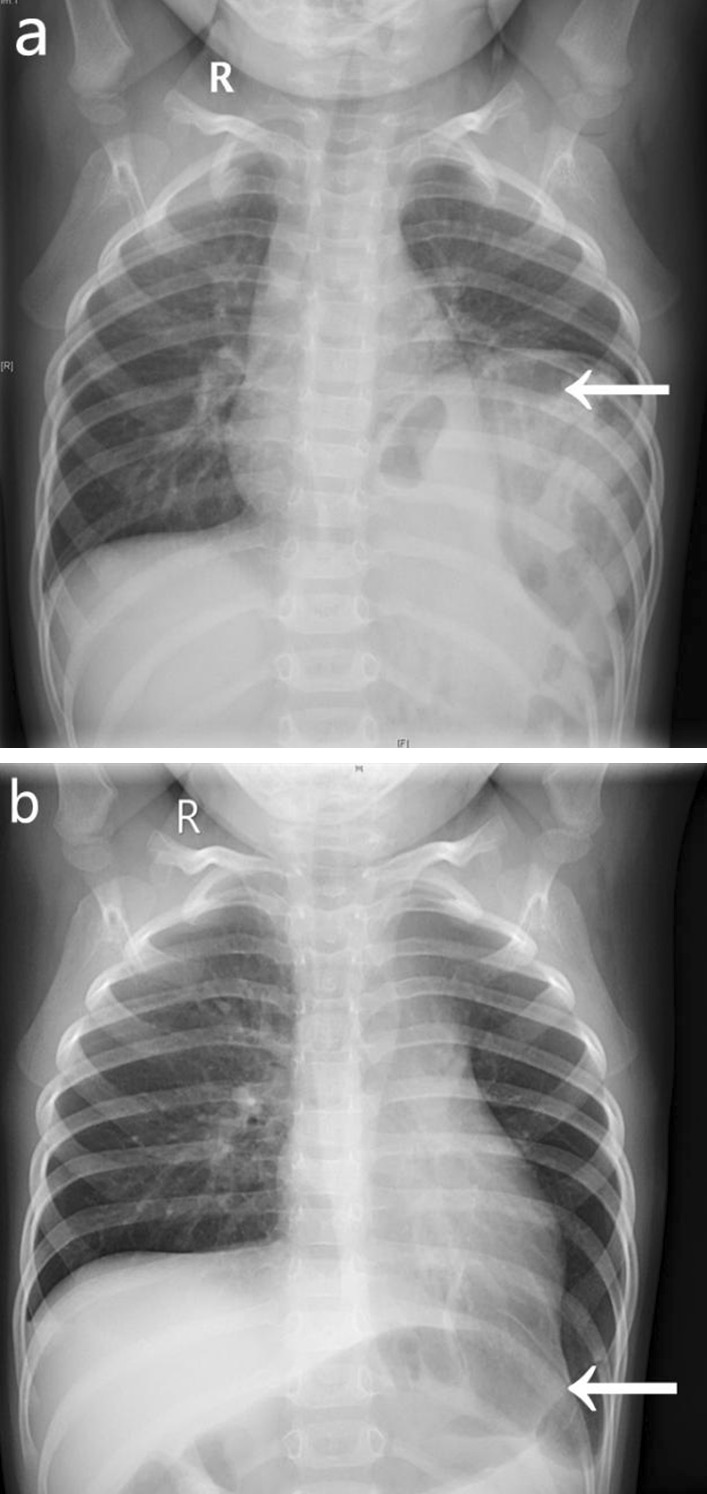
Preoperative chest radiograph of a child with CDE shows a raised left hemidiaphragm (**a**). A radiograph taken in the postoperative recovery period shows the left hemidiaphragm in the normal position (**b**)

The patients were followed radiologically on an annual basis to demonstrate the position of the diaphragm, and symptoms, if any, were also evaluated. In the open surgery group, 1 patient died due to respiratory distress after the operation. Almost all respiratory and digestive symptoms disappeared within 1 month after the operation, and no patients had any symptoms 3 years after surgery. After 1–9.5 years of follow-up, 107 patients had significantly improved preoperative symptoms. Eleven of the 17 children who did not undergo surgical treatment did not see a significant decrease in diaphragm position after 1–6 years of follow-up, and 6 patients were lost to follow-up. The comparison of chest radiographs before and after the operation is shown in Fig. 2.

## Discussion

CDE is characterized by incomplete muscle regeneration. Subsequent abnormally elevated diaphragm muscles cause abnormal movement of the affected hemidiaphragm during respiration. It can occur locally or affect the entire diaphragm. In this study, there were 90 males (72%) and 35 females (28%); 78 children (62.4%) had CDE on the right side, and 47 children (37.6%) had CDE on the left side. We observed that the incidence was higher in male children, and the incidence on the right side was higher than that on the left side. CDE can be associated with other developmental defects, and associated comorbidities include congenital hypoplastic lung, congenital heart disease, pectus excavatum, cleft palate, hypospadias, cryptorchidism, and congenital torticollis [4]. Seventy-seven patients in this group also had other malformations, and congenital heart disease (19, 15.2%) and congenital hypoplastic lung (16, 12.8%) were the main relevant abnormalities in this study. The above facts make it difficult to determine whether CDE is accompanied by other malformations or other malformations with this disease. Its numerous accompanying malformations suggest that the cause of teratology is difficult to explain with a single etiology and may be similar to the cause of other congenital malformations.

The main symptom of CDE is compression of the lower lobe of the lungs due to the increase in intra-abdominal organs. Compression can also cause the mediastinum to move on the healthy side, and the lung function of the healthy side can be reduced accordingly. In unilateral CDE, the lung capacity and total lung capacity are reduced by 20–30% [5]. Bilateral diaphragmatic eventration reduces lung function even more seriously, especially in the supine position [6]. The treatment principle of CDE is to restore the normal anatomical position and tension of the diaphragm. The method is to strengthen the weak diaphragm, and the goal is to maintain normal lung volume and lung ventilation. Whether asymptomatic patients need surgical correction has been controversial for a long time. In this group of 17 children who did not undergo surgical treatment, 11 patients received 1–6 years of follow-up, and a decrease in diaphragm position was not observed. Therefore, we believe that symptomatic children need timely surgical treatment. Yazici et al.'s study also considered symptomatic children, who usually require surgery [7]. Therefore, we believe that the indications for surgery are as follows: ① relative to the normal diaphragm position, the diaphragm is displaced upwards by 3 or more intercostals; ② diaphragm eventration causes obvious compression on the affected side of the lung and obvious shortness of breath, asthma and other respiratory distress symptoms; ③ frequent lung infections, hypoxemia, and even abnormal breathing exercises; and ④ during follow-up, the diaphragm continues to rise, and the eventration is aggravated.

The traditional treatment method of CDE is diaphragmatic plication performed either by laparotomy or thoracotomy. However, with the development of minimally invasive technology, thoracoscopy has gradually been applied in the treatment of CDE [8–10]. We believe that children with right diaphragm eventration and intrapulmonary malformation need to be corrected through the thoracotomy approach as the first choice because it is not affected by the intestinal canal, there is full exposure, there operation is easy, the phrenic nerve can be visualized, and postoperative intestinal paralysis can be reduced. Laparotomy is suitable for children with left diaphragmatic eventration, in cases where it is not possible to distinguish diaphragmatic eventration from diaphragmatic hernia, and when gastrointestinal malformation is considered. Because the heart is in the left chest, there is a high risk associated with thoracotomy. The use of a subcostal incision is conducive to hernia repair and the discovery of possible intestinal malformations. However, in the open group, we used thoracotomy in 4 children with diaphragmatic eventration on the left side and achieved satisfactory clinical results. Therefore, we believe that the choice of approach is mainly based on the characteristics of the patient’s diaphragmatic disease and surgeon familiarity with the approach. The preoperative diagnoses of 9 children in this group were unknown, and diaphragmatic hernia and other gastrointestinal tract malformations were found during the operation, so the choice of preoperative approach was particularly important. We resected the weak portions of the diaphragm via the thoracoabdominal route and sutured the diaphragm intermittently with nonabsorbable sutures to make the cut diaphragm imbricate in order to strengthen the weak area of the diaphragm. The advantage of this technique is that it increases the tension of the diaphragm to evenly distribute the tension throughout the repair area.

With the development of minimally invasive technology, thoracoscopy has gradually been used in the treatment of CDE. We compared the effects of open surgery and thoracoscopy in the treatment of CDE in children. The operation time, chest drainage time, postoperative mechanical ventilation time, postoperative hospital stay and postoperative CCU admission time in the thoracoscopy group were shorter than those in the open group, and the difference between the two groups was statistically significant (P < 0.05). We consider the following possible reasons. ① Thoracoscopic surgery adopts the three-hole method, which is less traumatic and less prone to bleeding. The recovery of children is faster after the operation. ② The technique of thoracoscopy requires advanced skill, and the operator and assistant cooperate with each other. ③ We used barbed wire to continuously suture without knots, which greatly shortens the operation time and is obviously better than open surgery.

In this group of 41 children without other thoracoabdominal malformations that need to be corrected, we used thoracoscopic diaphragm plication. Various techniques of diaphragmatic plication have also been employed. All techniques aim to reduce the abundant diaphragmatic surface and lower the diaphragmatic dome. Various suturing methods have been used, including interrupted horizontal mattress sutures, multiple parallel U sutures, figure-eight sutures, continuous running sutures, and endostaplers. Various nonabsorbable and also absorbable sutures have been used. We used barbed wire to suture the diaphragm from the outside to the inside in a continuous imbricated fashion to strengthen the diaphragm. Combined with the literature and our experience, compared with ordinary absorbable sutures, continuous suturing of the diaphragm with barbed wire has the following advantages. ① Starting from the second stitch, slippage is not easy after tightening the suture. One stitch is sewn to tighten one stitch, and no knot is needed during the suture process, which greatly shortens the operation time. ② The diaphragms were sutured continuously by barbed wire to make the diaphragms stretch evenly from the center in all directions. The tension distribution was uniform so that the movement of the diaphragms was more coherent. The diaphragms were not ischemic due to overtight suturing, and the suture did not relax to cause recurrence. ③ Barbed wire sutures are closes, have less bleeding, have a wireless knot, are absorbable, and have wireless knot reactions and residual suture. There is a view that continuous sutures may compromise suture safety, and loosening of the knot may affect the folding of the entire diaphragm, but there is no evidence to support this view [11]. Parlak et al. and others adopted the double-purse suture method to strengthen the diaphragm, achieving a better clinical effect [12]. The usual advantages of thoracoscopy, such as reduced postoperative pain, satisfactory appearance and rapid recovery, are also applicable in our surgery, which should be the preferred treatment for CDE.

## Conclusion

The clinical symptoms of congenital diaphragmatic eventration vary in severity. Patients with severe symptoms should undergo surgery. Both thoracoscopic diaphragmatic plication and traditional open surgery can effectively treat congenital diaphragmatic eventration, but compared with open surgery, thoracoscopic diaphragmatic plication has the advantages of a short operation time, less trauma, and a rapid recovery. Thus, thoracoscopic diaphragmatic plication should be the first choice for children with congenital diaphragmatic eventration.

## Data Availability

The datasets used and analyzed during the current study are available from the corresponding author upon reasonable request.

## Abbreviations

CDE: Congenital diaphragmatic eventration
ASD: Atrial septal defect
VSD: Ventricular septal defect
PDA: Patent ductus arteriosus
TAPVC: Total anomalous pulmonary venous connection
CCU: Coronary care unit
CT: Computed tomography
